# Capping Exposed Pulps

**Published:** 1889-07

**Authors:** 


### Capping Exposed Pulps.

In performing this delicate operation many practitioners apply oxyphosphate or oxychloride of zinc as a capping. This is fraught with many dangers well known to the dental specialist. Some claim that oxychloride of zinc, from its stimulating properties, will induce hyperactivity in the nutritive currents, thereby producing an excess of the pabulum which nourishes and organizes dentrine, but if used indiscriminately may result in congestion or death of the pulp. Phosphate of zinc is considered better by some, as it possesses less caustic properties, is more ductile, and possesses better edge strength. The objections to these materials may be removed by first placing a disc formed of gutta-percha, fitting loosely over the exposed pulp, filling the disc with oxide of zinc, formed into a paste by the addition of oil of cloves. Then place a small piece of asbestos felt over the disc preparatory to introducing the plastic material. In case of an extremely sensitive condition of the tubuli of the tooth a thorough "*whitewashing*" of cavity is recommended—*i. e.*, procure a small quantity of lime, mix with a thin solution of sandarac varnish, and apply to the walls of the cavity. This procures a thoroughly antiseptic lining to the walls of the cavity, and protects the tooth against thermal changes that play such sad havoc with the imprisoned pulp in cases of congestion.

